# Introduction to Modern EW Systems. Edited by Andrea De Martino, Artech House, 2012; 417 pages. Price: £119.00, ISBN 978-1-60807-207-1

**DOI:** 10.3390/s130101146

**Published:** 2013-01-17

**Authors:** Shu-Kun Lin

**Affiliations:** MDPI AG, Kandererstrasse 25, CH-4057 Basel, Switzerland; E-Mail: lin@mdpi.com

The following paragraphs are reproduced from the website of the publisher [1].

Master the latest electronic warfare (EW) techniques and technologies related to on-board military platforms with this authoritative resource. You gain expert design guidance on technologies and equipment used to detect and identify emitter threats, giving you an advantage in the never-ending chess game between sensor guided weapons and EW systems. This unique book offers you deeper insight into EW systems principles of operation and their mathematical descriptions, arming you with better knowledge for your specific design applications.

Moreover, you get practical information on how to counter modern communications data links which provide connectivity and command flow among the armed forces in the battlefield. Taking a sufficiently broad perspective, this comprehensive volume offers you a panoramic view of the various physical domains—RF, Infrared, and electronics—that are present in modern electronic warfare systems. This in-depth book is supported with over 280 illustrations and more than 560 equations.

**Table of Contents****Foreword****xi****Introduction****xiii****Acknowledgments****xiv****Chapter 1 Introduction to Electronic Warfare Scenarios****1**1.1Definitions and EW Role in the Military Field11.2Main Weapons Systems of Interest to EW31.2.1Artillery Systems61.2.2Missile Systems71.2.3Active Homing Missiles101.2.4Track via Missile Systems121.2.5Passive IR-Guided Missiles131.2.6Sea-Skimming Missiles131.2.7Antiradiation Missiles141.3EW in Symmetric Conflicts151.4EW in Asymmetric Conflicts20References21**Chapter 2 Evolution of Signal Emitters and Sensors****23**2.1Introduction232.2Sensor Electromagnetic Spectrum and Atmospheric Propagation242.3Radar Principles and Types262.3.1Radar Equation292.3.2Radar Structure322.3.3Radar Signal Processing Fundamentals372.3.4Automatic Detection502.3.5Pulse Compression552.3.6Surveillance Radars652.3.7LPI Radars662.3.8Pulse Doppler Radars752.3.9Tracking Radars832.3.10Synthetic Aperture Radars1012.3.11Bistatic Radars1042.4Communications1132.4.1Access Methods1152.4.2Digital Signaling1162.4.3Secure Communications1192.4.4Coding of Communication Signals1202.4.5Typical Military Communication Systems1232.5Satellite Navigation Systems1252.6Electro-Optical Thermal Imagers1292.6.1Minimum Resolvable Temperature1362.6.2IR Missile Seekers1392.6.3Missile Approach Warner1472.7Laser Radar Systems1472.7.1Laser Target Designation and Ranging1482.7.2Laser Radar Receivers1492.7.3Laser Radar Range Equation1492.7.4Target Detection153References154**Chapter 3 Electronic Warfare RF Band Sensor Systems****157**3.1Introduction1573.2EW Radar Band Sensors1583.2.1RWR Architecture1593.2.2ESM Architecture1603.2.3ELINT Architecture1613.3EW Sensor Sensitivity1613.3.1Conclusions1683.4Probability of Interception1683.5EW Radar Band Sensor Architectures1753.5.1Architecture of Past Generation Intercept Receivers1753.5.2Architecture of New EW Radar Band Sensors1843.5.3DSP Technologies1923.6Detection and Classification of LPI Radars1973.7Emitter Deinterleaving and Sorting2043.8Emitter Identification2063.9Communications ESM2083.9.1CESM2083.9.2COMINT2153.10SIGINT2163.11Conclusion217References220**Chapter 4 RF Direction-Finding and Emitter Location Techniques****221**4.1Introduction2214.2Amplitude Comparison DF Methods2224.3Phase Comparison Monopulse DF Measurement Methods2294.3.1Correlative Phase DF2324.4Time-Difference DF2384.5Emitter Location2444.5.1Triangulation2454.5.2Trilateration2474.5.3Frequency Difference on Arrival Passive Location Technique2514.5.4Inverse Passive Location2554.6Conclusion259References259**Chapter 5 Electronic Countermeasure Systems****261**5.1Introduction2615.1.1Typical RECM Requirements and Missions2635.1.2EW Radar Jamming Equation2645.2Radar ECM Architecture2675.3Digital Radio-Frequency Memory2725.3.1Phase-Sampled DRFMs2745.4Radar ECM Transmitters2765.5Chaff2895.6Communication ECM Systems2915.7Infrared ECM Systems2955.7.1Flares3005.8Conclusion302References302**Chapter 6 ECM Techniques and Sensor ECCMs****303**6.1Introduction3036.2ECM Principles and Techniques Used Against Surveillance Radars and Related ECCMs3046.2.1Frequency Agility in Transmission3046.2.2PRI Agility3046.2.3Ultralow Sidelobes3056.2.4Multiple-Sidelobe Cancellers3056.2.5Sidelobe Blanker3086.2.6Adaptive Arrays3106.2.7Noise Jamming3116.2.8False Targets3126.3ECM Principles and Techniques Used Against Tracking Radars and Related ECCMs3136.3.1Range Tracking Loop Deception3146.3.2RECM Techniques Used Against Radar Doppler Tracking3186.3.3RECM Techniques Used Against Radar Angle Measurement3206.4Conclusions About RECM Techniques3376.5ECM Principles and Techniques Used Against Communication Systems3386.5.1Noise Jamming3416.5.2Follower Jammer3436.6Conclusions About ECM Techniques345References346**Appendix A Signal Detection in Sensor Receivers****349**A.1Integration of Successive Radar Pulses354A.2Coherent Detection355References356**Appendix B Introductory Concepts of Estimation Theory****357**B.1Maximum Likelihood Function Estimator359B.2Least-Squares Method of Estimation360Reference361**Appendix C Antennas and Phased Array Antennas****363**C.1Antenna Types366C.2Array Antennas368References379**Appendix D Analog Modulation Methods****381**D.1Amplitude Modulation381D.2Angle Modulation381D.3Quadrature Modulation384Reference385**Appendix E Evaluation of BER Increase for Noise and CW Tone Jamming in Communication Systems Employing BFSK Modulation**387References392**List of Acronyms****393****About the Author****401**
